# Pediatric-Isolated Auricular Erythromelalgia: A Case Report

**DOI:** 10.1155/2012/854081

**Published:** 2012-09-13

**Authors:** Kelly Grandy, Gerard Corsten, Paul Hong

**Affiliations:** ^1^Faculty of Medicine, Dalhousie University, Halifax, NS, Canada B3H 4R2; ^2^IWK Health Centre and Division of Otolaryngology-Head and Neck Surgery, Dalhousie University, Avenue 5850/5980, P.O. Box 9700, Halifax, NS, Canada B3K 6R8

## Abstract

Erythromelalgia is a rare disorder that typically affects the skin of the feet, hands, or both, that is characterized by red skin, warmth, and a burning quality of pain. It usually affects both sides of the body, but may manifest unilaterally. Cooling of the affected areas usually results in symptom relief. We report a case of a young boy with erythromelalgia of the ears.

## 1. Introduction

Erythromelalgia is a rare condition, usually affecting the extremities, where the individual suffers from episodes of intense burning pain, erythema, warmth, and heightened sensitivity to warm temperatures [1, 2]. The pain is usually relieved by cold temperatures and frequent immersion into ice-cold water [2]. Relief of the pain with cold water is described in most to all of the cases of erythromelalgia and is considered pathognomonic [2, 3]. The symptoms are typically felt bilaterally, but occasionally may be unilateral, and are typically classified on a scale from mild to severe [1, 3]. The symptoms are exacerbated by heat, warm environments, exercise, and dependent positions, with flare-ups being episodic or active most of the time [2, 3].

There have been three types of erythromelalgia described in the literature, which include primary (familial or sporadic type), secondary (due to multiple causes such as a myeloproliferative disorder, autoimmune disease, neuropathic condition, diabetes, and drugs), and more recently a third type, which is associated with thrombocythemia [1–4].

Erythromelalgia had characteristically only been seen in the extremities affecting the feet and hands, with more extreme cases involving the head and neck, but more recently few cases of auricular erythromelalgia, involving only the ears, have been described in the literature [1, 2].

In the present paper, we describe a case of a young boy who was diagnosed with erythromelalgia of the ears.

## 2. Case Report

A 7-year-old boy was seen in the pediatric ear surgery clinic for evaluation of an unusual 2-year history of burning sensation and pain of his external ears. More specifically, there was a history of an intermittent sudden onset of burning sensation and erythema of both of his ears, lasting about 20 minutes. The patient reported experiencing approximately 7 of these episodes per day, which required immediate ice pack application to the ears to attain symptomatic relief.

There was no history of reported hearing difficulties or other otologic complaints. He did report one episode of an ear infection, which was treated with antibiotics in the past and this seemed to settle his external ear symptoms momentarily, but he shortly redeveloped the episodic burning of his ears.

Past medical history included being born one month premature and being the smaller fraternal twin. There were no issues at birth, and he has never required a prolonged hospital stay. He had no known drug allergies and there was no family history of any skin disorders.

**Figure 1:**
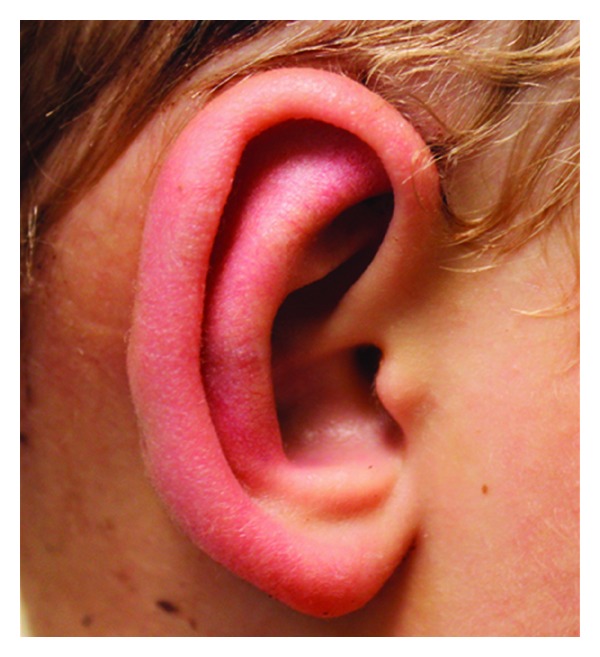
A photograph of the right ear showing mild erythema.

Examination revealed mild redness of the ears bilaterally, with no evidence of chondritis, cellulitis, or swelling (Figure 1). The ear exam was completely normal otherwise showing normal tympanic membranes with no evidence of any middle ear fluids. The external auditory canal was also normal. The rest of the head and neck exam, including examination of the cranial nerves and neurologic screen, was unremarkable.

Following the initial consultation, a diagnosis was not provided, and a referral was made to the pediatric dermatology clinic to investigate further and to assess for any potential cutaneous causes. After this referral, a diagnosis of erythromelalgia of the ears was made. Suggestions were made to try topical agents such as pramocaine hydrochloride or pramoxine to help the burning sensation, given their anesthetic or menthol components. Blood work, including inflammatory markers, was within normal limits. Cholesterol and liver enzymes were also normal.

The episodes gradually started to occur on a less frequent basis and the severity was also reduced. At his 16-month follow-up visit, the episodes had resolved.

## 3. Discussion

Isolated auricular erythromelalgia is a very rare disorder, especially in the pediatric population. A recent series reviewed 32 cases of generalized pediatric erythromelalgia, which was gathered over a 37-year period [5]. Of these 32 cases, 22 had been girls and 10 had been boys, with a range from 5 to 18 years of age. Symptoms seemed to be intermittent in 26 of these patients and constant in the other eight. Like in adults with this condition, extremities were most commonly affected: the feet were involved in all 32 cases and hands were involved in 47% of the patients. Interestingly, the ears were affected in only two of these patients. The mean age at diagnosis was 13.8 years, taking an average of 4.6 years to be properly diagnosed [5]. Overall, only three cases of isolated erythromelalgia involving only the ears were identified in the literature [1, 2, 6], indicating the unusual nature of the present case.

Erythromelalgia is a diagnosis of exclusion and no specific investigations have been recommended [1–4, 6]. Noninvasive vascular studies (e.g., laser Doppler flow) and neurophysiologic tests with autonomic reflex screening may be performed in some cases [5], but it is unclear how necessary they are in regards to the diagnosis and management of this disease.

Due to the paucity of erythromelalgia, the definitive diagnosis may be delayed in some cases (see below). Other clinical entities to consider are recurrent soft-tissue infections, reflex sympathetic dystrophy, peripheral neuropathy, Raynaud phenomenon, vasculitic diseases, and Fabry disease [5]. As mentioned above, the diagnosis of erythromelalgia is mainly clinical.

Histopathological studies from skin biopsies of individuals with primary erythromelalgia showed relatively subtle and nonspecific findings, mostly pertaining to changes in and around blood vessels [7, 8]. However, no vascular thrombi were noted in arteries and arterioles, as has been reported in biopsies from patients with erythromelalgia secondary to myeloproliferative disease [8]. The marked redness of the skin is likely caused by inflammation, consistent with the histological finding of superficial and deep perivascular inflammation with lymphocytes and endothelial cells [1]. On examination of nerve fiber density from these specimens, it was found that the epidermal nerve fiber count was below the 5th percentile for each of the sites in 81% of the cases [8]. Reduced nerve fiber density was also found around the dilated capillary loops in 75% of the patient specimens studied [8]. These findings are consistent with small fiber neuropathy, which is likely the cause of the painful burning sensation experienced by these patients.

 There is still much to learn about erythromelalgia. The symptoms and onset can vary greatly from person to person, from mild to severe, to gradual, or acute. There does not seem to be a universally effective treatment or therapeutic approach that works for everybody, which has been documented in many studies [1, 3–5]. It appears that most cases of erythromelalgia in children are not inherited, and thus are idiopathic [5]. In the past, depending on disease severity and location, this condition could become quite disabling and was considered more of a chronic disease. However, newer research has shown that erythromelalgia is a reversible condition in some patients [3]. One report described a case of a 58-year-old man who after eight years finally found a medication that resulted in sustained relief of his symptoms [2]. Specifically, mexiletine, a medication used in neuropathic pain, which works by decreasing the frequency and stability of neuron firing involved in nociceptive pain processing, was successful in symptom alleviation for this adult patient [2].

In the literature around pediatric erythromelalgia, the treatment results are inconclusive due to the low incidence and a large number of individuals being lost to long-term followup. Yet, there have been some reports of spontaneous resolution or gradual reduction of the frequency and severity of the symptomatic episodes [5]. Fortunately, this finding was also observed in our patient.
